# Supplementary figures and images for: The CCB-ID approach to tree species mapping with airborne imaging spectroscopy
Source: PeerJ. 2018 Oct 8;6:e5666. doi: 10.7717/peerj.5666 (PMC6181071; doi:10.7717/peerj.5666)

Model performance on test data using sample probabilities

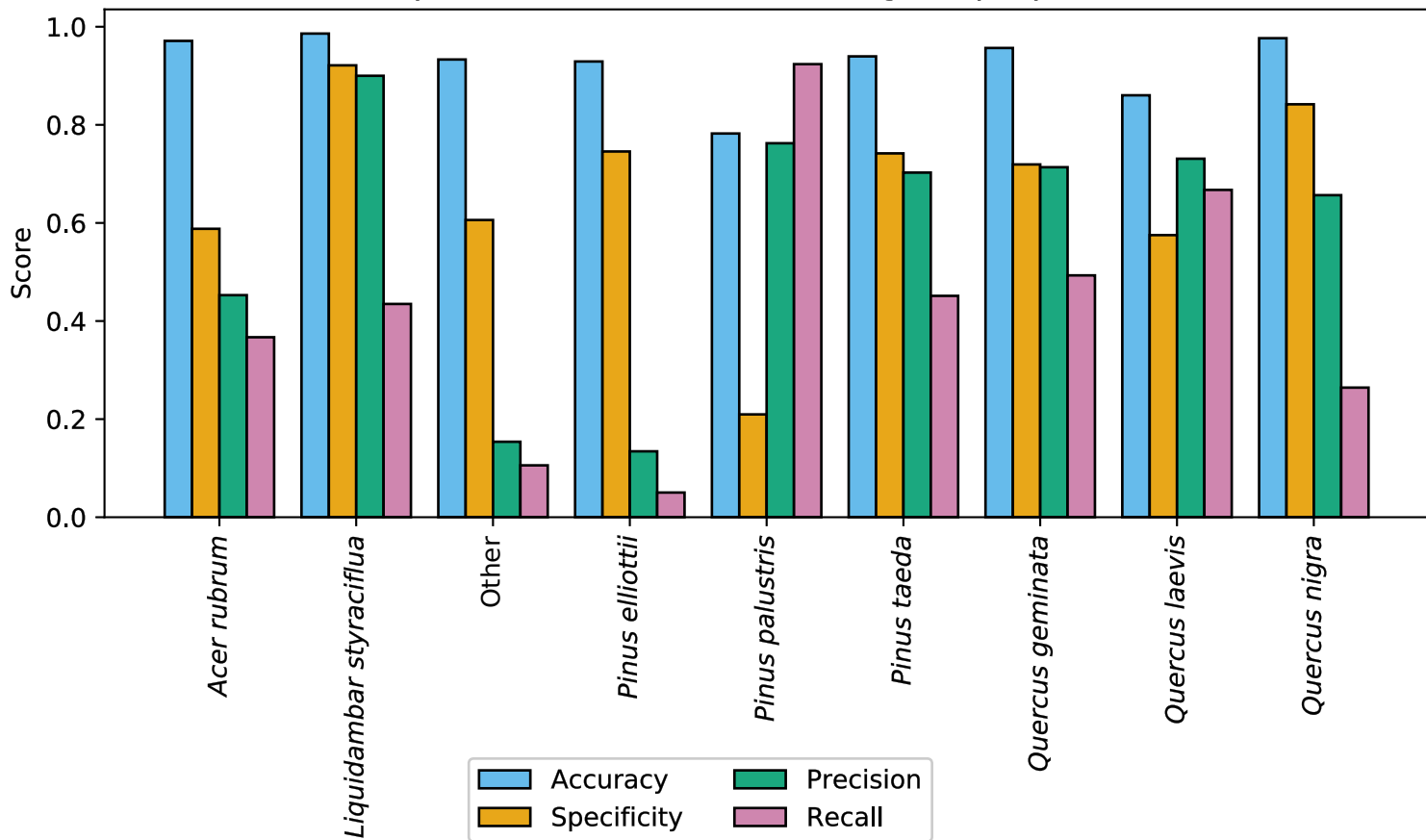

Supplement: Supplemental Information 1 — Per-species secondary performance metrics from the test data. These metrics were calculated using the prediction probability confusion matrix reported in Table S1. Low specificity scores for Pinus palustris, which do not appear in the binary classification results (Fig. 3, main text) reflect how it was frequently predicted at higher probabilities as a minority class. [file peerj-06-5666-s001.pdf]
