# Supplementary material for: The CCB-ID approach to tree species mapping with airborne imaging spectroscopy
Source: PeerJ. 2018 Oct 8;6:e5666. doi: 10.7717/peerj.5666 (PMC6181071; doi:10.7717/peerj.5666)
Supplement: Supplemental Information 2 — Prediction probability results of the CCB-ID model using the competition test data. Each cell contains the sum of prediction probabilities from all observed crowns per species. These data were used to generate Fig. S1. [file peerj-06-5666-s002.docx]

|  |  |  |  |  | **Predicted** |  |  |  |  |  |  |
| --- | --- | --- | --- | --- | --- | --- | --- | --- | --- | --- | --- |
|  | Species ID | Acer rubrum | Liquidambar stryaciflua | Other | Pinus elliottii | Pinus palustris | Pinus taeda | Quercus germinata | Quercus laevis | Quercus nigra | Total observed (TP) |
|  | Acer rubrum | **0.91** | 0.03 | 0.35 | 0.03 | 0.05 | 0.13 | 0.06 | 0.08 | 0.37 | 2 |
|  | Liquidambar stryaciflua | 0.03 | **0.90** | 0.03 | 0.01 | 0.01 | 0.00 | 0.01 | 0.00 | 0.01 | 1 |
|  | Other | 0.61 | 0.78 | **0.46** | 0.09 | 0.15 | 0.12 | 0.63 | 0.05 | 0.10 | 3 |
| **Observed** | Pinus elliottii | 0.02 | 0.02 | 0.04 | **0.27** | 0.92 | 0.42 | 0.13 | 0.14 | 0.05 | 2 |
|  | Pinus palustris | 0.45 | 0.24 | 1.43 | 4.32 | **64.17** | 3.01 | 1.65 | 7.79 | 0.94 | 84 |
|  | Pinus taeda | 0.12 | 0.07 | 0.72 | 0.24 | 0.57 | **3.53** | 0.44 | 0.22 | 0.08 | 6 |
|  | Quercus germinata | 0.04 | 0.02 | 0.26 | 0.10 | 0.33 | 0.07 | **2.84** | 0.29 | 0.03 | 4 |
|  | Quercus laevis | 0.16 | 0.06 | 1.21 | 0.31 | 3.27 | 0.52 | 0.37 | **16.81** | 0.29 | 23 |
|  | Quercus nigra | 0.23 | 0.00 | 0.02 | 0.01 | 0.03 | 0.02 | 0.00 | 0.03 | **0.65** | 1 |
